# Conservation Status of the Family Orchidaceae in Spain Based on European, National, and Regional Catalogues of Protected Species

**DOI:** 10.1155/2018/7958689

**Published:** 2018-01-30

**Authors:** Daniel de la Torre Llorente

**Affiliations:** Biotechnology-Plant Biology Department, Higher Technical School of Agronomic, Food and Biosystems Engineering, Universidad Politécnica de Madrid, 28140 Madrid, Spain

## Abstract

This report reviews the European, National, and Regional catalogues of protected species, focusing specifically on the Orchidaceae family to determine which species seem to be well-protected and where they are protected. Moreover, this examination highlights which species appear to be underprotected and therefore need to be included in some catalogues of protection or be catalogued under some category of protection. The national and regional catalogues that should be implemented are shown, as well as what species should be included within them. This report should be a helpful guideline for environmental policies about orchid's conservation in Spain, at least at the regional and national level. Around 76% of the Spanish orchid flora are listed with any figure of protection or included in any red list, either nationally (about 12–17%) or regionally (72%).

## 1. Introduction

The family Orchidaceae is widely represented in Europe, with 35 known genera, of which 25 are present in the Iberian Peninsula—26 including the Canary Islands—where the representation of this family is one of the most extensive ones, at least on an extratropical, equivalent level or only slightly lower than in other countries of the Mediterranean region. Taxonomic and floristic studies of this family in the Iberian Peninsula have been increasing slowly, though in a fragmented way over time. The first published quotes regarding the Orchidaceae family date back to 1861 [1, 2], and the first in-depth study of the family was conducted in 1887 [3]. Later, in 1930, a doctoral thesis examining the species in Spanish territory was published [4], but it was not until 1973 that a compendium comprising more or less all knowledge of Spanish orchids to date was published [5]. Since the 1960s, numerous French, Belgian, German, and English orchidologists have visited Spain and have extensively contributed, with the description of new taxa, to taxonomic and corological knowledge of this family. In addition, since 1970, herborization by numerous Spanish botanists has generated many studies, resulting in the development of abundant local, provincial, and regional catalogues of the Iberian territory, which contain new information concerning representatives of the Orchidaceae family, such as Granada [6, 7], Navarre [8], Portugal [9, 10], Albacete [11], Andalusia [12, 13], Balearic Islands [14, 15], Cuenca [16, 17], Extremadura [18, 19], Jaén [20], Burgos [21–23], Catalonia [24–26], Alicante [27, 28], Málaga [29], Almería [30], Galicia [31, 32], La Rioja [33], the Basque Country [34], Murcia [35], and Aragón [36]. The most updated and complete corological work that collects information on the distribution of Orchidaceae in the autonomous community of Madrid dates to 1994 [37]. Later works have helped to supplement such information [38].

Additional evidence of incipient interest in the Orchidaceae family is the publication of several European and Mediterranean orchid guidelines [39–46]. All this research work has generated an intense proliferation of names given to members of the Orchidaceae family in Spanish territories. However, recent works are helping to simplify the nomenclature complexity of existing synonymy for this family. These works include the entire family, certain subfamilies, tribes or subtribes [47–60], or more specific works at the level of certain genera like* Serapias *[61],* Dactylorhiza *[62, 63],* Nigritella *and* Gymnadenia* [64–66],* Anacamptis, Orchis, *and* Neotinea *[67–69],* Limnorchis *and* Platanthera* [70],* Chamorchis *and* Traunsteinera* [71], or* Ophrys* [72, 73].

The study of the taxa in this family, specifically its conservation and stability in landscapes, is essential for the stability of the vegetation and landscape [19]. Much of the representatives of the family Orchidaceae appear in areas with minimal processing and a certain environmental stability, although there may be human presence. This situation has facilitated the use of orchids as bioindicators in the environmental management of the territory in dehesas [74] and deciduous forests [75] and in the conservation of habitats.

## 2. Material and Methods

The nomenclature suggested by Flora iberica has been followed in this work for the family Orchidaceae [76].

The following catalogues and regulations have been checked.

At the* European level*, the following have been consulted:

- *Habitats Directive* [77]
- *Berne Convention* [78].

At the* national level*, the following have been consulted:

- *National Catalogue of Threatened Species* (CNEA) [79] and successive modifications and updates that affect the Orchidaceae family [80–82]
- *Law on Natural Heritage and Biodiversity* [83]
- *Red List of Spanish Vascular Flora *[84] and successive modifications and updates:
- *2008 Red List of Spanish Vascular Flora* [85]
- *2010 Red List of Spanish Vascular Flora, updated with the data of the Addendum 2010 to the Atlas and Red Book of the Vascular Threatened Flora* [86]
- *Atlas and Red Book of the Vascular Threatened Flora* [86–89].

At the* regional level*, the following have been consulted:

- *Plan of Recovery of Cypripedium calceolus *L*. in Aragón *[90]—the only regional plan for recovery of orchids in Spain
- *Plans for recovery and conservation of certain wild species and habitats protected in Andalusia* [91]
- *Red List of the Vascular Flora of Andalusia* [92]
- *Red List of the Vascular Flora of CAPV* (Comunidad Autónoma del País Vasco), thereafter Red List of the Basque Country [93].

Likewise, this study examined the seventeen corresponding regional catalogues (RC) of each of the seventeen autonomous Spanish communities:

1. Andalusia [94, 95]
2. Aragón [96–98]
3. Asturias [99]
4. Balearic Islands [100]
5. Basque Country [101–103]
6. Canary Islands [104, 105]
7. Cantabria [106]
8. Castilla-La Mancha [107, 108]
9. Castilla y León [109]
10. Catalonia [110]
11. Comunidad Valenciana [111]
12. Extremadura [112]
13. Galicia [113]
14. La Rioja [114]
15. Madrid [115]
16. Murcia [116]
17. Navarre [117].

The data obtained from the aforementioned catalogues were analysed, and the results of this analysis are presented in the Results of this report.

The taxa included in the CNEA were those species, subspecies, or populations of wild flora that require specific measures of protection. The category in which those taxa must be included is determined by considering threat factors facing the taxa throughout its natural distribution area inside the national territory, notwithstanding any potential mitigating or aggravating local circumstances of that threat [79]. As a rule, a species, subspecies, or populations of wild flora that require specific or special measures of protection and conservation must be included in regional catalogues. Besides, some regions include more specific criteria for their catalogues. For example, the RC of the Balearic Islands includes those taxa that require conservation measures because of their special interest or because the taxa are not included in the CNEA [100]; the RC of the Canary Islands includes those taxa that require specific measures of protection or that are interesting for Canarian ecosystems [105]; the RC of Extremadura, the RC of Galicia, and the RC of La Rioja include those taxa that require specific measures of protection attending to their rarity, singularity, representativeness, or exceptional nature [112–114]. The RC of Catalonia includes those taxa that are threatened in Catalonia and that require conservation measures according to their ecological and environmental values [110]. The RC of Castilla-La Mancha includes those taxa that are native to Castilla-La Mancha and maintain stable populations in the region, are subjected to threat factors, or are of special interest, thus requiring specific measures of protection [107]. The RC of Andalusia includes those taxa that deserve special attention and protection due to their scientific, ecological, or cultural value or due to their singularity, rarity, or degree of threat; it includes as well those taxa that are protected in different appendixes of international directives and agreements ratified by Spain [94]; the same criteria are expressed in the Law on Natural Heritage and Biodiversity [83].

## 3. Results and Discussion

### 3.1. At the European Level

*Cypripedium calceolus* L. and* Spiranthes aestivalis *(Poir.) Rich. are the two only orchid species included in the* Habitats Directive* whose distribution area includes Spain [77]. No orchid species whose distribution area includes Spain appear in the* Berne Convention *[78].

### 3.2. At the National Level

Although this report follows the nomenclature suggested by Flora iberica [76], which includes a total of 89 Iberian species belonging to 25 different genera, this study also adds four species of Canarian orchid flora not present in the Iberian orchid flora (adding the genus* Habenaria*, which is not present in the Iberian Peninsula):* Habenaria tridactylites* Lindl.,* Himantoglossum metlesicsianum *(W. P. Teschner) P. Delforge,* Orchis canariensis* Lindl., and* Serapias mascaensis* H. & G. Kretschner & Kreutz. There are also three species not listed in Flora iberica as individual species, but as a synonym of species already described above. However, the three species were identified as individual species after the publication of the corresponding volume of Flora iberica in which the family Orchidaceae is described [76]. These three species are as follows:

- *Dactylorhiza cantabrica* H.A. Pedersen: this species was named as* Dactylorhiza insularis *Ó. Sánchez & Herrero [76]; it was described as new species for Lugo in 2006 [118].
- *Orchis robusta* (Steph.) Gölz  & H. R. Reinhard: this species was described as* Orchis laxiflora *subsp.* robusta* Lam. [52] or as* Orchis palustris* Jacq. var.* robusta* T. Steph. [76]. It is now considered endemic of La Albufera de Mallorca (Balearic Islands) as a separate species according to several authors. Bateman and collaborators, in their molecular studies, treated it as a species next to* Orchis palustris* Jacq., but independent of it; they even proposed a combination of* O. robusta *within* Anacamptis robusta* (T. Steph.) Bateman [49]. Authors such as Delforge also consider* O. robusta* a different species of all of them [44]. However, Tyteca and Klein combine* O. robusta, A. robusta, *and* O. palustris* in a different genus with the name* Herorchis robusta* (T. Stephenson) Tyteca & Klein [60].
- *Serapias occidentalis *C. Venhuis & P. Venhuis: this species was included within* Serapias vomeracea *(Buró. Fil.) Briq. It has been proposed as a new species by hybridization of* Serapias vomeracea* subsp.* vomeracea *(Burm. fil.) Briq.* x Serapias cordigera* L. at Badajoz (Extremadura) [119].

Therefore, this work considers a total of 96 species present in Spain belonging to the Orchidaceae family.

Once the National Catalogue of Threatened Species [79] was revised, no orchid species were found; subsequent additions and modifications included* H. metlesicsianum* in the Danger of Extinction category (included as* B. metlesicsiana*) [80] and subsequently* C. calceolus* in the same category [81].

In the recent update of the CNEA in 2011 [82], the two species listed first (*H. metlesicsianum* and* C. calceolus*) have the same category of protection, and two more species are added in the List of Wild Species in Regime of Special Protection,* Orchis provincialis* Balb. and* S. aestivalis*.

*O. provincialis* presents a kind of Mediterranean distribution that restricts its presence mainly to the northern and western areas of the Iberian Peninsula.* S. aestivalis* is dispersed throughout almost all of the Iberian Peninsula, though more commonly found in the north and west and in specific habitats that are vulnerable to tampering, such as peat bogs, quagmires, reed beds, and wet heaths [76].

The Law of Natural Heritage and Biodiversity [83] implements the National Catalogue of Threatened Species [79], which includes two species of orchids,* C. calceolus*, within Appendix  II (Animal and Plant Species of Community Interest Whose Conservation is Necessary to Designate Special Areas of Conservation), and* S. aestivalis*, within Appendix  V (Animal and Plant Species of Community Interest Requiring a Strict Protection).

The categories of threat to species listed in Table 1 are those described by the International Union for the Conservation of Nature [120].

*Threatened Species*

  CR: Critically Endangered
  EN: Endangered
  VU: Vulnerable

*Not Threatened Species*

  NT: Near Threatened
  LC: Least Concerned
  DD: Data Deficient

Out of the 96 species of orchids present in the Iberian Peninsula, Balearic Islands, and Canary Islands, 16 species are included in the Red List of Spanish Vascular Flora (Table 1) [86], which were already included in the Red List of the Threatened Spanish Vascular Flora of 2008 [85]; five of these species are catalogued as not threatened species (i.e., NT, LC, or DD categories), which means that around 12–17% (depending on whether those five species are included) of the Spanish orchid flora are listed with some degree of threat nationwide, according to the 2010 Red List. Out of these 16 species, there are only two Canarian species—*H. metlesicsianum* and* O. canariensis.* So, if these two Canarian threatened species are omitted, there would be 14 species (or 9 species, if those five species mentioned above are not considered) with some degree of threat out of a total of 92 Iberian species (excluding the four species from the Canary Islands), that is, around 10–15% of the Iberian orchid flora.

#### 3.2.1. Threatened Species: Critically Endangered (CR)

At the national level, there are four species listed as Critically Endangered (CR), according to the 2010 Red List of Spanish Vascular Flora:* Corallorhiza trifida* Châtel.*, Epipogium aphyllum* Sw.*, O. robusta, *and* Orchis spitzelii* Saut. ex. W. D. J. Koch (Table 1) [86].

*C. trifida*. In the 2000 Red List of Threatened Vascular Flora of Spain, this species was listed as Endangered (EN), having gone to be classified as Critically Endangered (CR) in the 2008 Red List of Threatened Vascular Flora of Spain [85]; it went from having five 10 × 10 Km UTM coordinates in 2000 to only one 10 × 10 Km UTM in 2008. This species is still included in the 2010 Red List of Spanish Vascular Flora [86]. On the other hand, this species appears in the RC of Aragón (Danger of Extinction) (Table 1), where it currently holds the only recently confirmed population (Ordesa, Huesca, Aragón) [87] and in the RC of Catalonia (Danger of Extinction) (Table 1), where it is probably extinct, since the four populations that correspond to four 10 × 10 Km UTM coordinates have not been confirmed recently [87]. It should, therefore, be included in the CNEA and an urgent plan to recover such an endangered orchid should be developed.

*E. aphyllum*. This species is included in the 2010 Spanish Red List of Spanish Vascular Flora [86] and in the RC of Catalonia (Danger of Extinction) (Table 1), where its only population in the Pallars Sobirà of Lleida has not been confirmed recently; on the other hand, this species has two 10 × 10 Km UTM more, one in Sierra Cebollera (La Rioja) and the other one between Huesca and Navarre, in the Valley of Linza (Huesca, Aragón) and the Belagua Valley (Navarre) [87]; therefore, it is suggested that this species should be included in the RC of Aragón, RC of Navarra, and RC of La Rioja, where it is currently not included, most likely because these catalogues are relatively old, dating back to 1995, 1997, and 1998, respectively. Of course, this species should be included in the CNEA as well.

*O. robusta*. Although its description dates from 1976 [121], this species has been recently discontinued as a subspecies [44, 49], meaning that it is included only in the 2008 and 2010 Red Lists of Spanish Vascular Flora [85, 86]. It is endemic to the Balearic Islands and is found only in the Albufera of Mallorca (Balearic Islands). Therefore,* O. robusta* should be included in the CNEA as well as in the RC of Baleares.

*O. spitzelli*. Although this species is relatively widely distributed, with twenty-one 10 × 10 Km UTM coordinates, it currently only has a single 10 × 10 Km UTM recently confirmed in the Sierra del Cadí (Lleida, Catalonia). For this reason, this species is listed as Critically Endangered (CR) [85, 86] and it is only listed in the RC of Catalonia (Danger of Extinction) (Table 1), while it does not appear in the CNEA. Therefore, it is strongly suggested that this species be included in the CNEA.

These four species of orchids listed as Critically Endangered in Spain (CR) should have specific plans of recovery.

#### 3.2.2. Threatened Species: Endangered (EN)

There are two species classified as Endangered (EN) according to the 2010 Red List of Spanish Vascular Flora [86]:* C. calceolus *and* H. metlesicsianum*.

*C. calceolus*. This species is most likely, among all species of orchids included in the 2010 Red List of Spanish Vascular Flora, the species that is included in the most catalogues of protection, is the most studied, and has the most protection, since the threat to this species in the Iberian Peninsula was detected more than twenty years ago, being, along with* S. aestivalis,* the only two species protected in Spain that are included in the Habitats Directive [77]. In addition,* C. calceolus* and* H. metlesicsianum *are the only two species of Spanish orchids included in the CNEA in the category Danger of Extinction [82] and the only species, along with* S. aestivalis*, which are included in the Law of Natural Heritage and Biodiversity [83].* C. calceolus* is also included in the RC of Aragón (Table 1) and the RC of Catalonia (Table 1) and has appeared in the Atlas and Red Book of the Threatened Vascular Flora of Spain since 2004 [87]. This is the only orchid that has a Plan of Recovery in Spain [90]. Since the distribution of* C. calceolus* in Spain is restricted to Aragón and Catalonia, it does not seem necessary to include this species in any other catalogue, neither regional nor national.

*H. metlesicsianum*. This is one of the two Canarian species—along with* O. canariensis* (VU)—which are included in the 2010 Red List of Spanish Vascular Flora (Table 1) [86].* H. metlesicsianum*, whose distribution is restricted to Tenerife island (Canary Islands), has been included in the Atlas and Red Book of the Threatened Vascular Flora of Spain since 2004 [87] and it is included in the CNEA [80, 82] and the RC of the Canary Islands [104]; therefore, this species does not need to be included in any other protection catalogue.

#### 3.2.3. Vulnerable Species (VU)

The vulnerable orchid species include* D. cantabrica*,* Epipactis phyllanthes *G.E. Sm.,* O. canariensis*,* Serapias nurrica* Corrias and* S. occidentalis*.

There are sixteen species included in the Red List of Spanish Vascular Flora (Table 1) [86], and four of them are not included in any other national or regional catalogue. Three of these species are the above-mentioned* D. cantabrica, O. canariensis* and* S. occidentalis*. The fourth one is* O. robusta *(CR), which was discussed above.

These three species—*O. robusta*,* D. cantabrica,* and* S. occidentalis*—most likely are in the Red List and no other catalogue because they have recently been described as separate species [49, 118, 119];* O. canariensis* has not been described so recently but shows a relatively restricted distribution (Lanzarote and Fuerteventura; Canary Islands). Undoubtedly, the most troubling case is* O. robusta*, which is classified as Critically Endangered (CR); the other species, recent description or not, are not urgent in the short term, since they are classified as vulnerable (VU).

Nevertheless, and with the exception of* S. nurrica* and* E. phyllanthes*, none of the other three listed species as VU are found in any catalogue of protection; the recommendation would be to include those five species within the CNEA; in addition,* D. cantabrica*, which has been cited in O'Couto and Caurel (Lugo, Galicia), should be included in the RC of Galicia;* E. phyllanthes*, cited in Liencres (Santander, Cantabria) and Gorliz (Vizcaya, Basque Country), should be included in the RC of Cantabria and the RC of the Basque Country (although it is already included in the Red List of the Basque Country [93]);* O. canariensis*, endemic to the Canary Islands (Lanzarote and Fuerteventura) should be included in the RC of the Canary Islands;* S. occidentalis*, cited in Campo Lugar, Obanco, and Aljucén (Cáceres, Extremadura), should be included in the RC of Extremadura.

#### 3.2.4. Other Categories, Not Threatened Species: Near Threatened (NT), Least Concern (LC), and Data Deficient (DD)

As species catalogued as Near Threatened (NT), nationally, only* Serapias perez-chiscanoi* Acedo is included in the 2010 Red List of Spanish Vascular Flora [86] (Table 1); this species, in the 2000 Red List of Threatened Vascular Flora of Spain [84], had been classified as Vulnerable (VU), but the recent discovery of new populations of this species has led to its cataloguing as NT since 2008 [19, 85, 122]. This species is endemic to Portugal and Spain and more specifically to Extremadura (in Spain). This species is included in the RC of Extremadura as a species in Danger of Extinction [112]. Given that this regional catalogue is nine years older than the Spanish Red List, it explains that it seems not to be updated.

A second group of species under no threat at the national level but included in the 2010 Red List of Spanish Vascular Flora [86] are classified as Least Concerned (LC); in this group are* D. insularis*,* Dactylorhiza sulphurea* (Link) Franco, and* Nigritella gabasiana* Teppner & E. Klein.

*D. insularis*. This species is also included in the RC of Extremadura [112] as a species of Special Concern, since, without being regulated in any of the categories of greatest threat (Endangered, Sensitive to Alteration of Their Habitat, Vulnerable), it is worthy of particular attention on the basis of its scientific, ecological, and cultural value or by its singularity [112].

*D. sulphurea*. This species is included in the RC of Castilla y León [109] as one of Preferential Attention, since, without meeting the conditions to be attached to any of the categories of greatest threat (Endangered, Vulnerable, Sensitive to Alteration of Their Habitat, and Of Special Attention), the species is scarce in Castilla y León, presenting threatened or reduced populations, which could be affected by different disturbances or that are linked to habitats in regression [109]. Additionally, the species is classified as Vulnerable in the RC of the Basque Country (included as* Dactylorhiza markusii* (Tineo) H. Baumann & Künkele) [102]. It has also been recently included in the Red List of the Basque Country as Critically Endangered (CR) [93], which suggests that its status in the RC of the Basque Country as Vulnerable should be changed to Critically Endangered (CR). It was also included in the Red List of the Vascular Flora of Andalusia in the category of Data Deficient (DD) [92].

*N. gabasiana*. At the national level, this species was included as Vulnerable (VU) in the 2000 Red List of Threatened Vascular Flora of Spain [84], listed as Least Concern (LC) in the 2008 Red List of Threatened Vascular Flora of Spain [85], and remains so catalogued [86]. Additionally, it is classified as a Rare species in the RC of the Basque Country [102], and it is also included in the Red List of the Basque Country as Critically Endangered (CR) [93]; this fact does suggest that the status of this species in the RC of Basque Country as Rare should be changed to Critically Endangered (CR). It is also listed as Preferential Attention in the RC of Castilla y León, since, without meeting the conditions to be attached to any of the categories of greatest threat (Endangered, Vulnerable, Sensitive to Alteration of Their Habitat, and Of Special Attention), the species is scarce in Castilla y León, showing threatened or reduced populations and that they could be affected by different disturbances or are linked to habitats in regression [109].

Finally,* Gymnadenia odoratissima* (L.) Rich is classified at the national level as Data Deficient (DD) according to the 2010 Red List of Spanish Vascular Flora [86]. At the regional level, this species is listed as Preferential Attention in the RC of Castilla y León, since, without meeting the conditions to be attached to any of the categories of greatest threat (Endangered, Vulnerable, Sensitive to Alteration of Their Habitat, and Of Special Attention), it is scarce in Castilla y León, presenting threatened or reduced populations and that they could be affected by different disturbances or are linked to habitats in regression [109]. This species is catalogued as Vulnerable according to RC of Catalonia [110].

### 3.3. At the Regional Level

At the regional level, from the review of the corresponding seventeen regional catalogues of each autonomous Spanish community and the two existing regional red lists for Andalusia and the Basque Country [92, 93], the following results emerge.

Out of the 96 species of orchids existing in Spain, up to a total of 69 are included in any catalogue of regional protection or regional red list (including 12 of 16 protected species nationwide), that is, around 72%. Only four out of the sixteen species included in the 2010 Red List of Spanish Vascular Flora are not included in any regional catalogue nor any regional red list (*D. cantabrica, O. canariensis, O. robusta,* and* S. occidentalis*) (Table 1) [86]. If both, the species included in the national and regional catalogues are considered, that is, also including these four species, and then 73 species are included in some catalogue of protection, which means that 76% of the Spanish orchid flora are listed with some figure of legal protection.

In Tables 2–20 are the species of orchids included in each of the regional catalogues and the cataloguing as protected species for each autonomous community, indicating the piece of legislation that regulates it. These tables are as follows.

Table 2 specifies the species included in the regional catalogue that lists endangered species of Andalusia [94, 95]; Table 3 specifies those species that are included in the Red List of the Vascular Flora of Andalusia [92]; Table 4 is for Aragón [96, 98]; Table 5 is for Asturias [99]; Table 6 is for the Balearic Islands [100]; Table 7 is for the Basque Country [101–103]; Table 8 specifies those species that are included in the Red List of the Vascular Flora of the Basque Country [93]; Table 9 is for Canary Islands [104, 105]; Table 10 is for Cantabria [106]; Table 11 is for Castilla-La Mancha [107, 108]; Table 12 is for Castilla y León [109]; Table 13 is for Catalonia [110]; Table 14 is for Comunidad Valenciana [111]; Table 15 is for Extremadura [112]; Table 16 is for Galicia [113]; Table 17 is for La Rioja [114]; Table 18 is for Madrid [115]; Table 19 is for Murcia [116]; Table 20 is for Navarre [117].

The Red List of Andalusia (Table 3; [94, 95]) is not an official list with legislation but should serve as a basis for whether to include certain species in the RC of Andalusia (Table 2). Andalusia's RC has one notable absence,* D. sulphurea*. In addition,* Gennaria diphylla* (Link) Parl. and* Gymnadenia conopsea* (L.) R. Br. in W. T. Aiton, both listed as VU in the Red List of Andalusia, should be included as well in the RC of Andalusia [92].

The RC of Aragón [96], its update being relatively recent [97, 98] (Table 4), seems to collect the most endangered species in this region, some of them clearly nationally threatened, such as* C. trifida* and* C. calceolus* (Table 1) [86]; however, it would be convenient to include* E. aphyllum*, a species included in the 2010 Red List of Spanish Vascular Flora as a Critically Endangered species (CR) (Table 1) [86].

In the case of Asturias [99] (Table 5), despite its great biodiversity of flora, including orchids, the RC of Asturias does not include any species of the Orchidaceae family.

In the RC of the Balearic Islands [100] (Table 6), the presence of* S. nurrica *is notable, as it is also included in the 2010 Red List of Spanish Vascular Flora (VU) (Table 1) [86]. The inclusion of* O. robusta* in the regional catalogue should be pressing, as it is already included in the 2010 Red List of Spanish Vascular Flora as Critically Endangered (CR) (Table 1) [86].

The Red List of the Basque Country [93] (Table 8) includes the seven species of the RC of the Basque Country, generally with degrees of threat greater than those designated in the RC of the Basque Country: both* D. sulphurea* and* N. gabasiana* are listed as CR, and the other species listed as Rare in the RC of the Basque Country* (C. viride, E. palustris, *and* S. spiralis)* are catalogued in the Red List of the Basque Country as VU, as well as* Orchis italic* Por in Lam., which is still listed as VU. The seventh species,* Himantoglossum hircinum* (L.) Spreng., is catalogued as Special Interest in the RC of the Basque Country whereas in the Red List of the Basque Country it is catalogued as Near Threatened. The Red List of the Basque Country adds to these seven species five more:* E. phyllanthes*, which is listed as CR in the Basque Country and as VU at the national level according to the 2010 Red List of Spanish Vascular Flora (Table 1) [86];* Barlia robertiana* (R. J. Loisel) Greuter and* Orchis cazorlensis* Lacaita, both listed as CR;* Ophrys aveyronensis* (J. J. Wood) P. Delforge in P. Delforge & D. Tyteca as VU; and* Orchis papilionacea* L., which is classified as DD [93]. It would be advisable, therefore, to include at least* E. phyllanthes *within the RC of the Basque Country, a species cited in this community only at Gorliz (Bilbao). It would also be advisable to include the other four species, especially the two species listed as CR (*B. robertiana* and* O. cazorlensis*) and to a lesser extent* O. aveyronensis* (VU) and* O. papilionacea* (DD).

The RC of the Canary Islands [104, 105] (Table 9), which includes* H. metlesicsianum* (included as* B. metlesicsiana*. in the category Extinction Danger) [104], should be expanded with* O. canariensis*, included in the 2010 Red List of Spanish Vascular Flora as Vulnerable (VU) (Table 1) [86].

The RC of Cantabria [106] (Table 10) includes only one species,* Epipactis palustris* (L.) Crantz; however, it seems clear that* E. phyllanthes* should be included as well, as it is included in the 2010 Red List of Spanish Vascular Flora as a vulnerable species (VU) (Table 1) [86], cited only at Liencres (Santander, Cantabria).

Table 11 shows the species included in the regional catalogue of endangered species of Castilla-La Mancha [107, 108].

Table 12 shows the species included in the regional catalogue of endangered species in Castilla y León [109]. It is an extensive list, with eighteen species, three of which are included in the 2010 Red List of Spanish Vascular Flora:* G. odoratissima* (DD),* D. sulphurea *(LC), and* N. gabasiana* (LC) (Table 1) [86]; however,* O. spitzelii* is not included and perhaps it should be due to the few populations of this species in this region [22]; this species is included in the 2010 Red List of Spanish Vascular Flora as Critically Endangered (CR) (Table 1) [86].

Table 13 specifies those species included in the regional catalogue of endangered species in Catalonia [110]. Although it is not a very extensive listing, as it includes only a total of nine species, it is the regional catalogue that includes a greater number of species that are included in the 2010 Red List of Spanish Vascular Flora (Table 1) [86], with five species, most of them as Critically Endangered (CR:* C. trifida*,* E. aphyllum*, and* O. spitzelii*) or Endangered (EN:* C. calceolus*) at the national level, all of them catalogued as in Danger of Extinction according to the RC of Catalonia; only* G. odoratissima* (DD) is not threatened according to the Spanish Red List, classified as Vulnerable according to the RC of Catalonia. Therefore, and considering that it is one of the most recent catalogues, the regional catalogue of Catalonia seems to be very up to date. It should be noted that it is the only regional catalogue to include* O. spitzelii*, an endangered species according to the 2010 Red List of Spanish Vascular Flora (Table 1) [86], which is also exceptionally present in other autonomous communities such as Castilla y León or Aragón (not confirmed in Aragón).

Table 14 specifies the species included in the regional catalogue of endangered species of Comunidad Valenciana [111]. This is one of the most recent regional catalogues of endangered species of Spain; evidence of this recentness is its thoroughness, with a total of 41 species included (39 if synonyms are taken into account), although only* D. insularis* is included nationally in the 2010 Red List of Spanish Vascular Flora (LC) (Table 1) [86]. This catalogue appears to be updated as it is one of the most recent regional catalogues, and it lists a large number of species.

The Regional Catalogue of Extremadura [112] (Table 15) contains two of the species included in the 2010 Red List of Spanish Vascular Flora (Table 1) [86]—*D. insularis* and* S. perez-chiscanoi*; in the case of the latter, catalogued for Extremadura as Endangered and however listed nationally as Near Threatened (NT) according to the 2010 Red List of Spanish Vascular Flora (Table 1) [86], it seems clear that its status should change regionally to a degree of minor threat. It would be useful to include in this catalogue* S. occidentalis, *a species highly located in Extremadura, specifically at Campo Lugar, Obanco, and Aljucén (Cáceres). It should be listed as VU according to the 2010 Red List of Spanish Vascular Flora (Table 1) [86].

In the case of the Regional Catalogue of Galicia [113] (Table 16), with only one species,* S. aestivalis* (Vu), it should at least include* D. cantabrica*, catalogued nationally as VU according to the 2010 Red List of Spanish Vascular Flora (Table 1) [86] and cited only at O'Couto (Caurel, Lugo).

The Regional Catalogue of La Rioja [113] (Table 17) does not collect any species of the Orchidaceae family; it is recommended to include, at least,* E. aphyllum*, with one population in Sierra Cebollera and listed nationally as Critically Endangered (CR) according to the 2010 Red List of Spanish Vascular Flora (Table 1) [86].

The Regional catalogue of the Comunidad de Madrid is the most ancient of the many catalogues in Spain [115] (Table 18), including only two species—*Platanthera bifolia* (L.) Rich and* Neottia nidus-avis* (L.) Rich. The list of possible species to be included is long, with very specific or apparently extinct species in this autonomous community, as evidenced by recent studies [38]. These species include* Aceras anthropophorum *(L.) W. T. Aiton*, Anacamptis pyramidalis *(L.) Rich*, Barlia robertiana *(R. J. Loisel) Greuter*, Cephalanthera rubra *(L.) Rich,* D. insularis*,* Dactylorhiza sambucina* (L.) Soó*, G. conopsea, H. hircinum, Limodorum trabutianum* Batt.*, Listera ovata *(L.) R. Br. in W.T. Aiton*, Neotinea maculata* (Desf.) Stearn.*, Ophrys fusca *Link*, Orchis papilionacea *L.*, Serapias cordigera* L. and* Spiranthjes spiralis* (L.) Chevall.

Murcia's RC [116] (Table 19) seems to be up to date, since it is quite recent, dating from the year 2003, and it includes twelve species.

Opposite to Murcia's RC in terms of timeliness is that of Navarre's RC [117] (Table 20), which includes only one species,* Orchis papilionacea*. This catalogue should be reviewed, and it should include at least* E. aphyllum*, with one population at the Belagua Valley and that appears in the 2010 Red List of Spanish Vascular Flora as a species in critical Danger of Extinction (CR) (Table 1) [86].

### 3.4. Statistical Analysis

After conducting a joint analysis of the different regional catalogues, the following observations can be made.

Table 21 refers to the main genera that have been included in regional catalogues of protected species the most, indicating the number of times that each genus has been included in a regional catalogue and the number of species of this genus that have been included in a regional catalogue. The table also specifies the number of species for each genus at the national level following Flora iberica [76].

**Figure 1:**
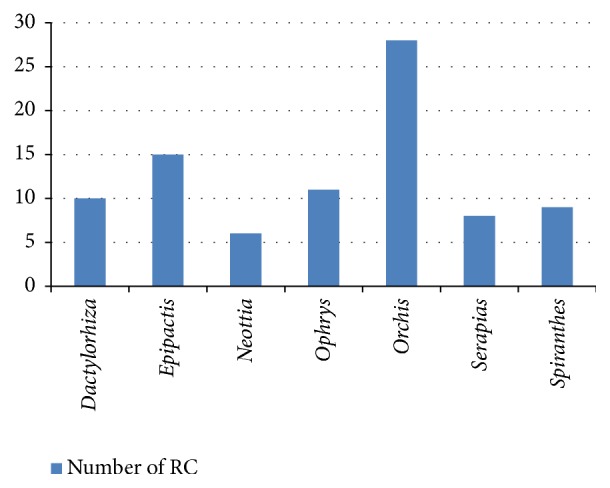
Genera mostly represented in regional catalogues (RC).

**Figure 2:**
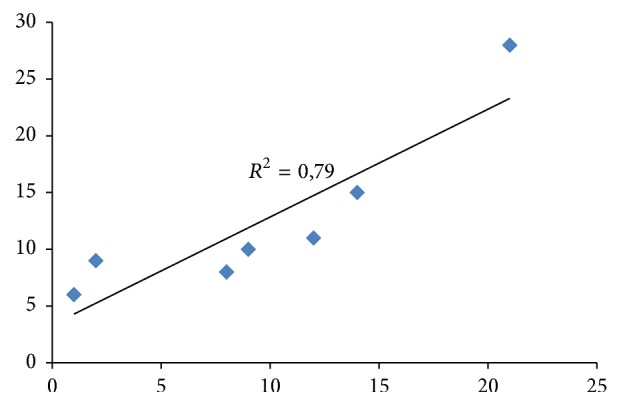
Relationship of regression between the number of species per genus and the number of regional catalogues (RC) in which each genus is included.

Figure 1 represents the genera whose species mostly have been included in the set of 17 regional catalogues. The genera that include large numbers of species and are therefore most likely to be present in many autonomous communities are also those that are included in a larger number of regional catalogues, stressing above all the genus* Orchis*, with 28 inclusions of different species in the regional catalogues, which precisely shows the greater number of species at the national level (21 species) (Table 21; Figure 1) [76]. In fact, if a correlation is made between the number of species that those genera have at the national level and the number of times that those genera have been included in any regional catalogue (represented by any kind of species that belong to that genus), the correlation is quite high (*R*^2^ = 0.79) (Figure 2).

At the opposite end, there are the genera* Neottia*, single grazing (*Neottia nidus-avis* (L.) Rich), and* Spiranthes*, with two species (*S. aestivalis* and* S. spiralis*), which are included in six and nine regional catalogues, respectively.

**Figure 3:**
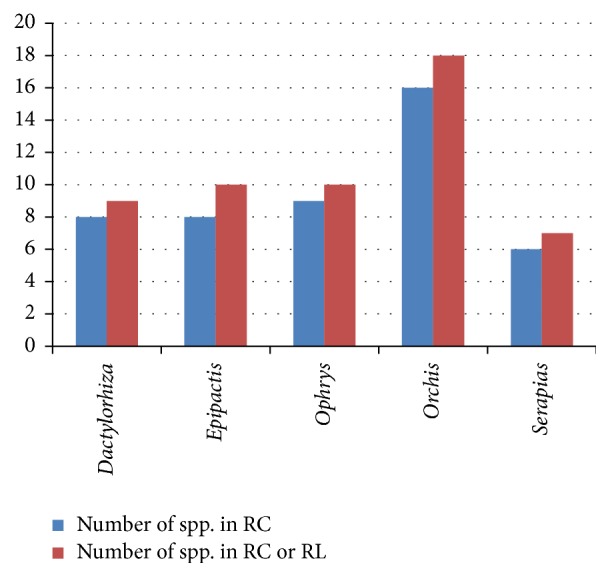
Genera with the highest number of species included in the regional catalogues. At the same time, the numbers of species included in regional catalogues and regional red lists are included.

In a complementary manner, in Figure 3 there are the genera with a larger number of species included in the regional catalogues, showing similar results to those in Figure 1 but excluding* Neottia* and* Spiranthes*, as they are only represented by one and two species, respectively.

For Figure 3, the results are more homogenous, and, except for* Orchis*, which remains standing with 16 species included in various regional catalogues, the other genera are represented through six to nine species in the different regional catalogues. This drop in the number of species is because several species are repeated in several catalogues, but there are really not so many different species, especially in the case of* Orchis* and* Epipactis*, featuring 28 and 15 appointments in different regional catalogues (Figure 1) corresponding to 16 and 8 species, respectively (Figure 3, Table 21). The correlation between the number of species per genus and the number of species that are included in regional catalogue per genus continues to present a high determination coefficient (*R*^2^ = 0.88; figure not shown). Likewise, if the number of times that a genus is included in any regional catalogue is correlated with the number of species of that genus included in regional catalogues, the correlation is quite high (*R*^2^ = 0.92; figure not shown), as expected.

**Figure 4:**
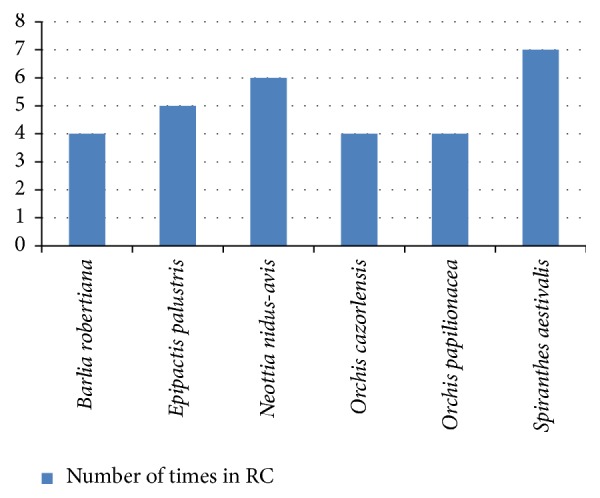
Species mostly included in regional catalogues (RC).

Similarly, Figure 4 represents the species that are included in the greatest number of regional catalogues, highlighting* S. aestivalis* and* N. nidus-avis*, found in seven and six regional catalogues, respectively. None of the six most cited species in regional catalogues (four to seven cites) are included in the 2010 Red List of Spanish Vascular Flora [86], which is indicative of their wide national distribution, although in certain autonomous communities they are scarce; however,* S. aestivalis* is included in the CNEA [82].

Table 22 constitutes a compendium of the results after analysing the global set of 17 regional catalogues. The table indicates, for each one of these regions, the law by which its regional catalogue was created or its most recent modification (and therefore the year), the number of listed species, and if, based on the results mentioned above, an urgent modification is considered; it also includes the species most urgent to include in each catalogue and its conservation status if it exists, according to the Red List of Spanish Vascular Flora [86] (Table 1).

As evident in Table 22, there is no clear relationship between how recent each regional catalogue is and the number of protected species included in it (*R*^2^ = 0.1; figure not shown); nevertheless, it is quite obvious for some regional catalogues that seem obsolete, as in the cases of Madrid [115], Asturias [99], Navarre [117], La Rioja [114], and Basque Country [103], with antiquities from 11 to 21 years, with no update since then. These regional catalogues clearly require updating, and they must include some species to protect, as in the case of* E. aphyllum* in Aragón, La Rioja, and Navarre, or multiple species that appear necessary to include in the catalogue of Madrid according to De la Torre & Gamarra [38], which in fact is the oldest regional catalogue of Spain; however, there are also quite recent catalogues that need to be updated in a broad way, such as the cases of Andalusia [94], the Canary Islands [105], Cantabria [106], and Galicia [113].

However, if the correlation is made between the antiquity of the catalogue and the number of species suggested to include, in this case, an acceptable correlation is obtained (*R*^2^ = 0.38; figure not shown), amounting to *R*^2^ = 0.57 if the curious case of Andalusia mentioned above is not taken into account (figure not shown). Although this regional catalogue dates from 2012, it is suggested to include at least four more species based on the Red List of the Vascular Flora of Andalusia [92]. For this regression, ten species are taken as the number of species to be included in the regional catalogue of Madrid, according to De la Torre and Gamarra [38]; in the case of Asturias, considering that the region has a more recent catalogue, six species have been taken as the number of species to be included in that regional catalogue. These results indicate that there is a direct correlation between the antiquity of the regional catalogue and the number of species to be included.

A fact that must be considered when making such correlations is that not all the Spanish regions have the same floristic richness and that, logically, those regions with more variable and more sensitive habitats tend to house vulnerable species and will be more likely to protect a greater number of species. Such is the case of the regions hosting the country's major mountain ranges, such as regions of Northern Spain and Cantabrian and Pyrenean (Galicia, Asturias, Cantabria, Basque Country, Navarre, and Catalonia) and the mountainous regions of the Centre-North (Castilla y León, Madrid) or South (Andalusia), where on average there are more than 16 genera of orchids, as opposed to other regions with a low diversity of orchids (Murcia, Aragón) [123].

## 4. Conclusions

There are no orchid species whose distribution area includes Spain that are included in the Berne Convention [78], and there are only two species included in the Habitats Directive [77]:* C. calceolus*, which is the species that is best protected at a legal level, and* S. aestivalis.*

Out of the 96 species of the Spanish orchid flora considered in this review, up to 73 species are included in any catalogue of protection; 16 are in the 2010 Red List of Spanish Vascular Flora (five of them not endangered) [86], and 69 are included in any catalogue of regional protection or regional red list (including 12 of the 16 species included in the aforementioned red list); that is, around 76% of the Spanish orchid flora are listed with any figure of protection or included in any red list, either nationally (about 12–17%) or regionally (72%). Only four out of the sixteen species included in the 2010 Red List of Spanish Vascular Flora are not included in any regional catalogue although they should be (Table 1, [86]).

The National Catalogue of Threatened Species (CNEA) lists a total of four species:* H. metlesicsianum* (Endangered) [80, 82],* C. calceolus* (Endangered) [81, 82], and* O. provincialis* and* S. aestivalis* (both as Wild Species in Regime of Special Protection) [82]. It is suggested that, in successive revisions of the CNEA, the other 14 species included in the 2010 Red List of Spanish Vascular Flora should be included—except for* C. calceolus* (EN) and* H. metlesicsianum* (EN), which are already included [86] (Table 1). Moreover, it is strongly suggested to include those species listed as Critically Endangered (CR) and Vulnerable (VU) and to a lesser extent the other species listed as Near Threatened (NT) and as Least Concern (LC) and those listed as Data Deficient (DD). In addition, the elaboration of specific plans of recovery for those species listed as Critically Endangered (CR) is suggested.

The genera mostly included in regional catalogues and which have a greater number of species in regional catalogues are precisely those genera that have a higher number of species at the national level:* Orchis, Epipactis, Ophrys, Dactylorhiza,* and* Serapias*; moreover,* Spiranthes* and* Neottia*, despite being bispecific and single grazing, respectively, are within the genera most included in regional catalogues. These latter two genera represent the two species most often included in regional catalogues:* S. aestivalis* and* N. nidus-avis*, which appear in seven and six regional catalogues, respectively.

Only three out of the seventeen regional catalogues of endangered species do not seem to need an immediate update: the RC of Catalonia [110], the RC of Comunidad Valenciana [111], and the RC of Murcia [116].

It is remarkable that the RC of Catalonia [110] is the one that lists the most species that are included in the Red List of Spanish Vascular Flora [86], a total of five species:* C. trifida* (CR),* E. aphyllum* (CR),* O. spitzelii* (CR),* C. calceolus* (EN), and* G. odoratissima* (DD).

The other fourteen regional catalogues would need to be modified to a greater or lesser extent, highlighting the RC of Madrid [115], which is the oldest of them all and probably the most obsolete; it is believed that at least ten species would need to be included [38]. The RC of Asturias is also quite old [99] and does not feature any orchid species. The regional catalogues of Andalusia [94] and the Basque Country [102], mainly thanks to the red lists of these two autonomous communities [92, 93], need to be incorporate at least four and five additional species, respectively (Table 22).

Several regional catalogues should also be highlighted for their remarkable absences: The RC of Aragón [98] should be modified to include* E. aphyllum* (CR) and perhaps* O. spitzelii* (CR) (its presence not confirmed); the RC of the Balearic Islands [100] should be modified to include* O. robusta* (CR), a species endemic to this autonomous community; the RC of the Canary Islands [105] should include* O. canariensis* (VU), a species endemic to this autonomous community; the RC of Extremadura [112] should include* S. occidentalis* (VU), which is endemic to Extremadura; the RC of Galicia [113] should include* D. cantabrica*, endemic to this autonomous community.

The main species that are listed in the 2010 Red List of Spanish Vascular Flora [86] and that would need to be included in several regional catalogues are the following:

 
*Orchis spitzelli* (CR): this species should be included in the regional catalogue of Castilla y León.
 
*Epipogium aphyllum* (CR): this species should be incorporated into three regional catalogues: Aragón, La Rioja, and Navarre.
 
*Epipactis phyllanthes *(VU): this species should be included in two regional catalogues: Cantabria and Basque Country.

## Figures and Tables

**Table 1:** The sixteen species included in *2010 Red List of Spanish Vascular Flora* (RL) [86] following IUCN threat categories [120]. It is shown if they are included in any regional catalogue (RC) and the threat category within them, as well as if they are included in any other catalogue of protected species.

	Threat category in RL	Regional catalogues (RC) and threat category	Other catalogues
*Corallorhiza trifida* Châtel.	CR (EN)^*∗*^	RC of Aragón (Danger of Extinction) RC of Catalonia (Danger of Extinction)	
*Cypripedium calceolus* L.	EN	RC of Aragón (Danger of Extinction) RC of Catalonia (Danger of Extinction)	CNEA (Danger of Extinction) Law Nat. Her. Biodiv. (Danger of Extinction) Habitats Directive Aragón Recuperation Plan
*Dactylorhiza cantabrica* H. A. Pedersen	VU		
*Dactylorhiza insularis* (Som.) O. Sánchez & Herrero	LC	RC of Extremadura (Special Interest)	
*Dactylorhiza sulphurea* (Link) Franco	LC	RC of Castilla y León (Preferential Attention)	RL Basque Country (CR) RL Andalusia (DD)
*Epipactis phyllanthes* G. E. Sm.	VU		RL Basque Country (CR)
*Epipogium aphyllum* Sw.	CR	RC of Catalonia (Danger of Extinction)	
*Gymnadenia odoratissima* (L.) Rich	DD (VU)^*∗*^	RC of Catalonia (Vulnerable) RC of Castilla y León (Preferential Attention)	
*Himantoglossum metlesicsianum* (W. P. Teschner) P. Delforge	EN	RC of Canary Islands (Interest for Canarian Ecosystems)	CNEA (Danger of Extinction)
*Nigritella gabasiana* Teppner & E. Klein	LC (VU)^*∗*^	RC of Catalonia (Vulnerable) RC of Castilla y León (Preferential Attention)	RL Basque Country (CR)
*Orchis canariensis* Lindl.	VU		
*Orchis robusta* (Steph.) Gölz & H. R. Reinhard	CR		
*Orchis spitzelii* Saut.ex W. D. J. Koch	CR	RC of Catalonia (Danger of Extinction)	
*Serapias nurrica* Corrias	VU	RC of Balearic Islands (Vulnerable)	
*Serapias occidentalis* C. & P. Venhuis	VU		
*Serapias perez-chiscanoi* Acedo	NT (VU)^*∗*^	RC of Extremadura (Danger of Extinction)	

^*∗*^Species included in *2000 Red List of Spanish Vascular Flora* (VV.AA, 2000) with another threatened category.

**Table 2:** Species included in the regional catalogue (RC) of Andalusia (DEC 23/2012).

RC of Andalusia (LAW 8/2003; DEC. 23/2012)	
*Neottia nidus-avis* (L.) Rich	Vulnerable
*Ophrys atlantica* Munby	Vulnerable
*Ophrys speculum *Link subsp.* lusitanica* O. Danesch & E. Danesch	Vulnerable (before as in Danger of Extinction)
*Spiranthes spiralis* (L.) Chevall.	Wild species in Protection Regime

**Table 3:** Species included in the Red List of the Vascular Flora of Andalusia [92].

Red List of Andalusia	
*Dactylorhiza markusii* (Tineo) H. Baumann & Künkele = *D*. *sulphurea* (Link) Franco	DD
*Dactylorhiza sulphurea* (Link) Franco^*∗*^	DD
*Epipactis lusitanica* D. Tyteca	DD
*Epipactis microphylla* (Ehrh.) Sw.	DD
*Gennaria diphylla *(Link) Parl.	VU
*Gymnadenia conopsea* (L.) R. Br. in W.T. Aiton	VU
*Listera ovata* (L.) R. Br. in W.T. Aiton	DD
*Neottia nidus-avis* (L.) Rich^*∗∗*^	EN
*Ophrys atlantica*Munby^*∗∗*^	DD
*Ophrys speculum *Link subsp.* lusitanica* O. Danesch & E. Danesch^*∗∗*^	DD
*Orchis palustris* Jacq.	DD
*Platanthera algeriensis* Batt. & Trab.	DD
*Spiranthes spiralis* (L.) Chevall.^*∗∗*^	DD

^*∗*^Species included in the *2010 Red List of Spanish Vascular Flora* (Table 1) [86]. ^*∗∗*^Species included in the RC of Andalusia.

**Table 4:** Species included in the regional catalogue (R.C.) of Aragón (DEC. 49/1995; ORD. 2004/03/04).

RC of Aragón (DEC. 49/1995; ORD. 4/03/2004; DEC. 181/2005)	
*Corallorhiza trifida *Châtel.^*∗*^	In Danger of Extinction
*Cypripedium calceolus *L.^*∗*^	In Danger of Extinction
*Ophrys riojana *C. E. Hermos. = *O. sphegodes* Mill.	Sensitive to Habitat Alteration
*Orchis simia* Lam.	Vulnerable

^*∗*^Species included in the *2010 Red List of Spanish Vascular Flora* (Table 1) [86].

**Table 5:** Species included in the regional catalogue (RC) of Asturias (DEC. 65/1995).

RC of Asturias (DEC. 65/1995)	
None	

**Table 6:** Species included in the regional catalogue (RC) of Balearic Islands (DEC. 65/1995).

RC of Balearic Islands (DEC. 75/2005)	
*Cephalanthera rubra* (L.) Rich.	Vulnerable
*Gymnadenia conopsea* (L.) R. Br. in W.T. Aiton	Vulnerable
*Neottia nidus-avis *(L.) Rich.	Vulnerable
*Orchis cazorlensis* Lacaita	Vulnerable
*Orchis palustris* Jacq.	Vulnerable
*Serapias nurrica *Corrias^*∗*^	Vulnerable

^*∗*^Species included in the *2010 Red List of Spanish Vascular Flora* (Table 1) [86].

**Table 7:** Species included in the regional catalogue (RC) of Basque Country (ORD. 3471/1998).

RC of Basque Country (ORD. 3471/1998)	
*Coeloglossum viride* (L.) Hartm.	Rare
*Dactylorhiza markusii *(Tineo) H. Baumann & Künkele* = D*. *sulphurea *(Link) Franco^*∗*^	Vulnerable
*Epipactis palustris* (L.) Crantz	Rare
*Himantoglossum hircinum* (L.) Spreng.	Special Interest
*Nigritella gabasiana *Teppner & E. Klein^*∗*^	Rare
*Orchis italica *Poir. in Lam.	Vulnerable
*Spiranthes aestivalis *(Poir.) Rich	Rare

^*∗*^Species included in the *2010 Red List of Spanish Vascular Flora* (Table 1) [86].

**Table 8:** Species included in the Red List of Basque Country [93].

Red List of Basque Country	
*Barlia robertiana *(R. J. Loisel) Greuter^*∗∗*^	CR
*Coeloglossum viride *(L.) Hartm.	VU
*Dactylorhiza sulphurea *(Link) Franco^*∗*^	CR
*Epipactis palustris* (L.) Crantz	VU
*Epipactis phyllanthes *G. E. Sm.^*∗∗∗* ^	CR
*Himantoglossum hircinum* (L.) Spreng	NT
*Nigritella gabasiana *Teppner & E. Klein^*∗*^	CR
*Ophrys aveyronensis *(J. J. Wood) P. Delforge in P. Delforge & D. Tyteca^*∗∗*^	VU
*Orchis cazorlensis *Lacaita^*∗∗*^	CR
*Orchis italica *Poir. in Lam.	VU
*Orchis papilionacea *L.^*∗∗*^	DD
*Spiranthes aestivalis *(Poir.) Rich	VU

^*∗*^Species included in the 2010 Red List of Spanish Vascular Flora (Table 1) [86]. ^*∗∗*^Species not included in the RC of Basque Country.

**Table 9:** Species included in the regional catalogue (RC) of Canary Islands (DEC. 151/2001; LAW 4/2010).

RC of Canary Islands (DEC. 151/2001; LAW 4/2010)	
*Himantoglossum metlesicsianum *(W. P. Tesch.) P. Delforge^*∗*^	Interest for Canary Ecosystems (before as in Danger of Extinction)

^*∗*^Species included in the *2010 Red List of Spanish Vascular Flora* (Table 1) [86].

**Table 10:** Species included in the regional catalogue (RC) of Cantabria (DEC. 120/2008).

RC of Cantabria (DEC. 120/2008)	
*Epipactis palustris* (L.) Krantz	In Danger of Extinction

**Table 11:** Species included in the regional catalogue (R.C.) of Castilla-La Mancha (DEC. 33/1998; DEC. 200/2001).

R.C of Castilla-La Mancha (DEC. 33/1998; DEC. 200/2001)	
*Aceras anthropophorum *(L.) W. T. Aiton	Special Interest
*Coeloglossum viride *(L.) Hartm.	Special Interest
*Dactylorhiza incarnata *(L.) Soó	Vulnerable
*Dactylorhiza sambucina *(L.) Soó	Vulnerable
*Dactylorhiza *spp.	Special Interest
*Epipactis distans *Arv.-Touv.	Special Interest
*Epipactis palustris *(L.) Crantz	Special Interest
*Gymnadenia conopsea *(L.) R. Br. in W. T. Aiton	Special Interest
*Himantoglossum hircinum* (L.) Spreng.	Special Interest
*Listera ovata* (L.) R. Br. in W. T. Aiton	Special Interest
*Neottia nidus-avis *(L.) Rich	Special Interest
*Ophrys insectifera* L.	Vulnerable
*Platanthera *spp.	Special Interest
*Serapias cordigera* L.	Special Interest

**Table 12:** Species included in the regional catalogue (RC) of Castilla y León (DEC. 63/2007).

RC of Castilla y León (DEC. 63/2007)	
*Barlia robertiana* (R.J.Loisel) Greuter	Preferential Attention
*Dactylorhiza sulphurea* (Link) Franco^*∗*^	Preferential Attention
*Epipactis fageticola *(C.E. Hermos.) Devillers-Tersch. & Devillers	Preferential Attention
*Epipactis microphylla *(Ehrh..) Sw.	Preferential Attention
*Epipactis palustris *(L.) Crantz	Preferential Attention
*Epipactis tremolsii *Pau	Preferential Attention
*Gymnadenia odoratissima *(L.) Rich^*∗*^	Preferential Attention
*Nigritella gabasiana *Teppner & E. Klein^*∗*^	Preferential Attention
*Ophrys insectifera* L.	Preferential Attention
*Orchis cazorlensis* Lacaita	Preferential Attention
*Orchis conica* Willd.	Preferential Attention
*Orchis pallens* L.	Preferential Attention
*Orchis papilionacea* L.	Preferential Attention
*Orchis provincialis* Balb. ex. Lam. & D.C	Preferential Attention
*Platanthera algeriensis *Batt. & Trab.	Vulnerable
*Platanthera chlorantha* (Custer) Rchb. in Mössler	Preferential Attention
*Pseudorchis albida* (L.) Á. Löve & D. Löve	Preferential Attention
*Spiranthes aestivalis* (Poir.) Rich	Preferential Attention

^*∗*^Species included in the *2010 Red List of Spanish Vascular Flora* (Table 1) [86].

**Table 13:** Species included in the regional catalogue (RC) of Catalonia (DEC. 172/2008).

RC of Catalonia (DEC. 172/2008)	
*Coeloglossum viride *(L.) Hartm.	Strictly Protected
*Corallorhiza trifida* Châtel. ^*∗*^	In Danger of Extinction
*Cypripedium calceolus *L.^*∗*^	In Danger of Extinction
*Epipogium aphyllum *Sw.^*∗*^	In Danger of Extinction
*Gymnadenia odoratissima *(L.) Rich^*∗*^	Vulnerable
*Ophrys catalaunica *(O. Danesch & E. Danesch.) Soca* = O.bertolonii *Moretti subsp*. catalaunica *(O. Danesch & E. Danesch.) Soca	Strictly Protected
*Orchis palustris *Jacq.	Vulnerable
*Orchis spitzelii *Saut. ex W. D. J. Koch^*∗*^	In Danger of Extinction
*Spiranthes aestivalis* (Poir.) Rich	Vulnerable

^*∗*^Species included in the *2010 Red List of Spanish Vascular Flora* (Table 1) [86].

**Table 14:** Species included in the regional catalogue (RC) of Comunidad Valenciana (DEC. 70/2009).

RC of Comunidad Valenciana (DEC. 70/2009)	
*Aceras anthropophorum *(L.) W. T. Aiton	Watched species
*Barlia robertiana* (R. J. Loisel) Greuter	Protected species not catalogued
*Cephalanthera damasonium *(Mill.) Druce	Watched species
*Coeloglossum viride* (L.) Hartm.	Vulnerable
*Dactylorhiza fuchsii* (Druce) Soó	Watched species
*Dactylorhiza incarnata* (L.) Soó	Vulnerable
*Dactylorhiza insularis* (Som.) O. Sánchez & Herrero^*∗*^	Vulnerable
*Dactylorhiza maculata* (L.) Soó	Protected species not catalogued
*Dactylorhiza sambucina *(L.) Soó	Watched species
*Epipactis atrorubens *Hoffm. ex Besser	Watched species
*Epipactis distans*	Watched species
*Epipactis fageticola*	Vulnerable
*Epipactis palustris* (L.) Crantz	Protected species not catalogued
*Epipactis rhodanensis* Gévaudar & Robatsch	Watched species
*Epipactis tremolsii *Pau	Watched species
*Goodyera repens* (L.) R. Br. in W. T. Aiton	Watched species
*Gymnadenia conopsea* (L.) R. Br. in W. T. Aiton	Watched species
*Limodorum trabutianum* Batt.	Watched species
*Listera ovata *(L.) R. Br. in W. T. Aiton	Protected species not catalogued
*Neottia nidus-avis* (L.) Rich	Protected species not catalogued
*Ophrys castellana *Devillers-Tesch. & Devillers* = O. sphegodes *Mill.	Protected species not catalogued
*Ophrys incubacea *Bianca* = O. sphegodes *Mill.	Protected species not catalogued
*Ophrys sphegodes* Mill.	Watched species
*Orchis cazorlensis *Lacaita	Protected species not catalogued
*Orchis collina *Banks & Sol. ex. Russell	Vulnerable
*Orchis conica* Willd.	Vulnerable
*Orchis coriophora *L. subsp. *martrinii* (Timb.-Lagr.) Nyman	Watched species
*Orchis fragrans* (Pollini) K. Richt.* = O. coriophora *L. subsp*. fragrans *(Pollini) K. Richt.	Protected species not catalogued
*Orchis italica *Poir. in Lam.	Watched species
*Orchis langei* K. Richt.	Watched species
*Orchis papilionacea *L.	In Danger of Extinction
*Orchis picta* (Loisl.) K. Richt. = *O. morio* L. subsp. *picta* (Loisl.) K. Richt.	Watched species
*Orchis purpurea* Huds.	Vulnerable
*Orchis ustulata* L.	Protected species not catalogued
*Platanthera bifolia* (L.) Rich	Protected species not catalogued
*Platanthera chlorantha* (Custer) Rchb. in Mössler	Protected species not catalogued
*Serapias lingua* L.	Vulnerable
*Serapias parviflora* Parl.	Protected species not catalogued
*Serapias strictiflora* Welw. ex Veiga	Vulnerable
*Spiranthes aestivalis *(Poir.) Rich	In Danger of Extinction
*Spiranthes spiralis* (L.) Chevall.	Watched species

^*∗*^Species included in the *2010 Red List of Spanish Vascular Flora* (Table 1) [86].

**Table 15:** Species included in the regional catalogue (RC) of Extremadura (DEC. 37/2001).

RC of Extremadura (DEC. 37/2001)	
*Cephalanthera rubra *(L.) Rich	Special Interest
*Dactylorhiza insularis *(Som.) O. Sánchez & Herrero^*∗*^	Special Interest
*Limodorum trabutianum *Batt.	Vulnerable
*Neottia nidus-avis *(L.) Rich	Vulnerable
*Ophrys dyris *(Maire) Soó in G. Keller Schltr. & Soó *= O*. *fusca *Linksubsp*. dyris *(Maire) Soó in G. Keller Schltr. & Soó	Special Interest
*Orchis italica *Poir. in Lam.	Special Interest
*Orchis langei *K. Richt.	Special Interest
*Orchis papilionácea *L.	Special Interest
*Serapias perez-chiscanoi *Acedo^*∗*^	In Danger of Extinction
*Spiranthes aestivalis *(Poir.) Rich	Special Interest

^*∗*^Species included in the *2010 Red List of Spanish Vascular Flora* (Table 1) [86].

**Table 16:** Species included in the regional catalogue (RC) of Galicia (DEC. 88/2007).

RC of Galicia (DEC. 88/2007)	
*Spiranthes aestivalis* (Poir.) Rich	Vulnerable

**Table 17:** Species included in the regional catalogue (R.C.) of La Rioja (DEC. 59/1998).

R.C. of La Rioja (DEC. 59/1998)	
None	

**Table 18:** Species included in the regional catalogue (RC) of Madrid (DEC. 18/1992).

RC of Madrid (DEC. 18/1992)	
*Platanthera bifolia* (L.) Rich	Vulnerable
*Neottia nidus-avis *(L.) Rich	Vulnerable

**Table 19:** Species included in the regional catalogue (RC) of Murcia (DEC. 50/2003).

RC of Murcia (DEC. 50/2003)	
*Aceras anthropophorum *(L.) W.T. Aiton	Vulnerable
*Barlia robertiana *(R.J.Loisel) Greuter	Vulnerable
*Cephalanthera rubra *(L.) Rich	Vulnerable
*Dactylorhiza elata* (Poir.) Soó	Vulnerable
*Epipactis cardina *Benito & C.E. Hermos.	Special Interest
*Himantoglossum hircinum* (L.) Spreng.	Vulnerable
*Listera ovata *(L.) R. Br. in W.T. Aiton	Vulnerable
*Ophrys incubacea * Bianca = *O. sphegodes* Mill.	Special Interest
*Orchis cazorlensis *Lacaita	Vulnerable
*Orchis purpurea* Huds.	Vulnerable
*Serapias lingua* L.	Vulnerable
*Serapias parviflora* Parl.	Vulnerable

**Table 20:** Species included in the regional catalogue (RC) of Navarre (DEC. 94 /1997).

RC of Navarre (DEC. Foral 94/1997)	
*Orchis papilionacea *L.	Sensitive to Habitat Alteration

**Table 21:** Number of species by genus in Spain [76], number of times that they are included in regional catalogues (RC) by genus, and number of species included in regional catalogues (RC) by genus.

	Number of spp./genus	Number of times in RC/genus	Number of spp. included in RC/genus
*Orchis*	21	28	16
*Epipactis*	14	15	8
*Ophrys*	12	11	9
*Dactylorhiza*	9	10	8
*Serapias*	8	8	6
*Spiranthes*	2	9	2
*Neottia*	1	6	1

**Table 22:** Number of species included in each regional catalogue, indicating in each case the most recent legislation that regulates it, as well as the need of an urgent modification, the minimum species to include in it, and their figure of protection—if it exists—according to the 2010 Red List of Spanish Vascular Flora [86] (Table 1).

Regional catalogue	Number of spp.	Needs modification?	Species to include
Andalusia (DEC. 23/2012)	4	YES	*Dactylorhiza sulphurea *(Link) Franco (LC) *Gennaria diphylla *(Link) Parl. *Gymnadenia conopsea *(L.) R. Br. in W.T. Aiton
Aragón (DEC. 185/2005)	4	YES	*Epipogium aphyllum *Sw. (CR)
Asturias (DEC. 65/1995)	0	YES	Undetermined
Balearic Islands (DEC. 75/2005)	6	YES	*Orchis robusta *(Steph.) Gölz & H.R. Reinhard (CR)
Basque Country (ORD. 3471/1998; ORD. 3901/2003)	7	YES	*Barlia robertiana* (Lois.) Greuter (CR) *Epipactis phyllanthes* G.E. Sm. (VU) *Orchis cazorlensis* Lacaita *Orchis papilionacea *L. *Ophrys aveyronensis* (J.J. Wood) P. Delforge in P. Delforge & D. Tyteca
Canary Islands (LEY 4/2010)	1	YES	*Orchis canariensis* (VU)
Cantabria (DEC. 120/2008)	1	YES	*Epipactis phyllanthes* G.E. Sm. (VU)
Castilla-La Mancha		NO	—
(DEC. 33/1998; DEC. 200/2001)			
Castilla y León (DEC. 63/2007)	18	YES	*Orchis spitzelii* Saut ex.W.D.J.Koch (CR)
Catalonia (DEC. 172/2008)	9	NO	—
Com. Valenciana (DEC. 70/2009)	41	NO	—
Extremadura (DEC. 37/2001)	10	YES	*Serapias occidentalis* C. & P. Venhuis (VU) *Serapias perez-chiscanoi* Acedo (NT)
Galicia (DEC. 88/2007)	1	YES	*Dactylorhiza cantabrica* H.A.Peders. (VU)
La Rioja (DEC. 59/1998)	0	YES	*Epipogium aphyllum* Sw. (CR)
Madrid (DEC. 18/1992)	2	YES	Undetermined
Murcia (DEC. 50/2003)	12	NO	—
Navarre (DEC. F. 94/1997)	1	YES	*Epipogium aphyllum *Sw. (CR)

## Additional Points

The findings of this work summarize the legislative knowledge about orchid conservation at several levels for Spain and should be a helpful guideline for environmental policies about orchid's conservation in Spain, at least at the regional and national level. These results will be presented to the Spanish Society for Biological Conservation of Plants (SEBiCoP).
